# Smoking cessation and smokefree environments for tuberculosis patients in Indonesia-a cohort study

**DOI:** 10.1186/s12889-015-1972-2

**Published:** 2015-07-02

**Authors:** Tara Singh Bam, Tjandra Yoga Aditama, Chen-Yuan Chiang, Rubaeah Rubaeah, Acep Suhaemi

**Affiliations:** International Union Against Tuberculosis and Lung Disease, Robinson Road, Singapore; Directorate General Disease Control and Environmental Health, Ministry of Health, Jakarta, Indonesia; International Union Against Tuberculosis and Lung Disease, Paris, France; Division of Pulmonary Medicine, Department of Internal Medicine, Wan Fang Hospital, Taipei Medical University, Taipei, Taiwan; Bogor City Health Office, Bogor, Indonesia; No Tobacco Community, Bogor, Indonesia

**Keywords:** Smoking, Cessation, Smokefree home, ABC-approach, Tuberculosis

## Abstract

**Background:**

Research indicates that smoking substantially increases the risk of tuberculosis (TB), delay in diagnosis, failure of TB treatment and death from TB. Quitting smoking is one of the best ways to prevent unwanted outcomes. Exposure to secondhand smoke increases the risks of both TB infection and development of active TB disease among children and adults. TB patients who smoke in the home are also placing their families at a greater risk of TB infection. It is very important to keep homes smokefree. The present study assessed the implementation and effectiveness of an intervention that promotes smoking cessation and smokefree environments for TB patients.

**Methods:**

All consecutive new sputum smear-positive TB patients (aged ≥15 years old) diagnosed and registered in 17 health centres between 1 January 2011 and 31 December 2012 were enrolled. The ABC (A=ask, B=brief advice, C=cessation support) intervention was offered for 5 to 10 minutes within DOTS services at each visit. Smoking status and smokefree environments at home were assessed at the first visit, each monthly follow up and at month six. Factors associated with quitting were analysed by univariable and multivariable analysis

**Results:**

Of the 750 TB patients registered, 582 (77.6 %) were current smokers, 40 (5.3 %) were ex-smokers and 128 (17.1 %) were never smokers. Of the 582 current smokers, 66.8 % had quit smoking at month six. A time from waking to first cigarette of >30 minutes, having a smokefree home and the display of “no smoking” signage at home at month six were significantly associated with quitting. Of the 750 TB patients, 86.1 % had created a smokefree home at six month follow-up compared with 18.5 % at baseline. All 80 health facilities were 100 % tobacco-free at the end of 2012 compared with only 52 (65 %) when the intervention began in March 2011.

**Conclusions:**

Brief advice of 5–10 minutes with minimal cessation support at every visit of TB patients resulted in high quit rates and higher awareness of adverse health effects of secondhand smoke exposure, which led patients to make their homes smokefree and health providers to make health care tobacco-free.

## Background

Tobacco smoking has rarely been mentioned among the challenges identified in tuberculosis (TB) control. The adverse association between smoking and TB is largely overlooked by clinicians treating TB [1]. In a large epidemiological study, 50 % of deaths from TB among Indian men was attributed to smoking [2]. A South Korean study demonstrated that current cigarette smokers had a 40 % increased risk of incident TB and were 55 % more likely to die of TB as compared with nonsmokers [3]. World Health Organization (WHO) reported that more than 20 % of global TB incidence may be attributable to smoking [4]. WHO and the International Union Against Tuberculosis and Lung Disease (The Union) published a Monograph on TB and Tobacco Control examining the associations between active and passive smoking and various TB outcomes including infection, occurrences of the disease, mortality, treatment outcomes and relapse after treatment [5].

The Union’s Lung Health Scientific Section established a working group on smoking cessation in TB patients in December 2009 at the World Lung Conference (WLC) in Cancun, Mexico to pilot a simple cost-effective approach that integrated TB and tobacco control measures within the existing health services. The working group consisted of governmental and non-governmental organisations from Bangladesh, Bhutan, Benin, Brazil, India, China, Indonesia, Nepal, South Africa, Pakistan, and Japan, Union members from different regions and countries, and staff of WHO [6]. This group’s efforts led to the development of the ABC (A=ask, B=brief advice, C=cessation support) approach outlined in The Union’s guide *Smoking Cessation and Smokefree Environments for Tuberculosis Patients* published in 2010 [7]. The ABC approach was piloted within the regular Directly Observed Treatment Short Course (DOTS) system in Bangladesh, Benin, Brazil, China, India, Indonesia, Mongolia, and South Africa.

We assessed the implementation and effectiveness of the ABC smoking cessation approach for TB patients and the establishment of smokefree environments in health care facilities and the TB patients’ homes in Indonesia. We report the findings of this assessment.

## Methods

This was a cohort study assessing a field based intervention.

### Settings

The burden of TB remains enormous in Indonesia. In 2012, there were an estimated 460,000 (185 per 100,000) incident cases of TB and 67,000 patients died from this disease. A total of 328,824 new and relapse TB cases were notified in 2012 [8]. Tobacco use is the leading cause of disease and premature death in Indonesia. WHO estimates that smoking kills 235,000 Indonesians annually and secondhand smoke takes another 25,000 lives. Smoking is largely unregulated and 61.4 million (36.1 %) of adults currently use tobacco. Smoking prevalence has increased among males to 67.4 % in 2011 from 53.4 % in 1995 [9]. The intervention was piloted in Bogor city, which had a population of 800,000 in 2012 [10], a smoke-free policy that was established in 2010 and 25 government health centres providing primary health services.

### Study population

Of the 25 health centres that provided DOTS services, 17 satisfied the criteria for piloting the intervention: 1) providing a written commitment to join the intervention, 2) having attended the initial training on smoking cessation and smoke-free environments in 2010, and 3) having registered at least 5 new sputum smear-positive TB cases in 2010. All consecutive new sputum smear-positive TB patients (aged ≥15 years old) diagnosed and registered in the 17 health centres between 1 January 2011 and 31 December 2012 were enrolled in the intervention. They were recruited within 7 days of commencement of anti-TB treatment.

### Establishing 100 % tobacco-free health care facilities

**Fig. 1:**
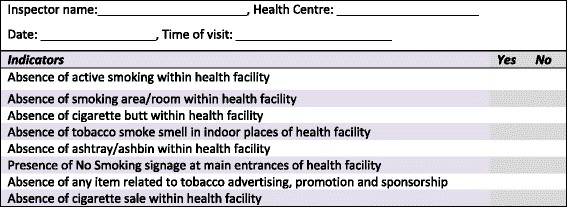
Checklist used to monitor tobacco free health care facility in Bogor city

We obtained written commitment from the Director of the City Health Office and the Head of each participating health centre to create tobacco-free services. Tobacco-free was defined as the absence of tobacco advertisement, promotion and sponsorship, the absence of any indoor or outdoor active smoking in the health facility, and the display of “No smoking” signage at the main entrance of the facility and removal of smoking-related accessories and items, such as ashtrays, smoking areas and cigarette butts. A total of 80 health facilities (including 17 DOTS health centres) participated in the tobacco-free initiative. An ABC orientation programme was conducted for all health staff at the health centres. Compliance of the 80 health facilities with their tobacco-free health care policy was assessed twice a year by the City Health Office and No Tobacco Community (civil society based in Bogor) using a standard checklist (Fig. 1). Assessment was usually conducted during the busiest hours.

### Establishing ABC smoking cessation intervention

**A = Ask** about smoking behaviour of TB patients via a face-to-face interview was conducted by health staff at each visit. At zero month of anti-TB treatment, they were asked: i) Do you smoke? ii) Have you smoked at all-even a puff-in the last three months? iii) Does anyone smoke inside your home? At all follow-up visits they were asked: i) Have you smoked at all-even a puff-in the last two weeks? ii) Does anyone smoke inside your home?

**B = Brief advice** which included personalised and general information was offered immediately at the DOTS clinic by the health staff at each visit. For smokers, personalised information consisted of 1) quitting smoking now you can recover properly from TB, and 2) as soon as you quit, your coughing and sputum will decrease. General advice for smokers and non-smokers included 1) smoking and secondhand smoke is very harmful for your and your family’s health; it causes diseases such as cancer, heart disease, chronic obstructive pulmonary disease, asthma, childhood pneumonia, and 2) to improve your and your family’s health, please quit smoking (for smokers) and do not allow anyone to smoke inside the home.

**C = Cessation support** was provided along with brief advice at each visit. Patients were advised to i) tell family, friends, and colleagues that they are quitting; ii) remove smoking accessories from home and workplaces; and iii) make their home smokefree and avoid secondhand smoke, and they were given health education leaflets, pamphlets and “No smoking” signage to display at home.

### Creating smokefree environments at home

Exposure to secondhand smoke was assessed and monitored as described above under ABC sections. TB patients were asked at zero month and at all follow up visits whether anyone was allowed to smoke inside the home, and whether “No smoking” signage was displayed at the main entrance of their home. Information obtained from the patient was verified with a family member that had accompanied the patient to the health centre. A telephone call was made to the patients’ closest family member to confirm that the home environment was smokefree if the patient had attended the health centre unaccompanied by family members.

### Monitoring and evaluation

A standard smoking cessation intervention card and register were adapted from The Union Guide 2010 (7). Information included in the smoking cessation intervention card were: age, sex, time from waking to first cigarette, as well as elements related to the intervention, such as frequency of ABC offered, smoking status, whether anyone smokes inside the home, confirmation of quit status by a family member and display of no smoking signs at home. The information was recorded from baseline to month six. ABC was offered for 5 to 10 minutes within the usual DOTS services. Information was updated on a monthly basis when patients were given ABC. Information related to TB diagnosis and treatment was obtained from the TB register, which is available at each health centres. Information from the smoking cessation intervention card was transferred to the register on a quarterly basis. Staff from the health centres also made random visits to some patients’ homes to see whether they had created smokefree environments at home. A standard checklist (Fig. 1) was used to monitor the tobacco-free status of the health care facilities. Quitting status and exposure to SHS were assessed by patients self-reporting during the period of anti-TB treatment and validated at month 6 through interviews (face-to-face or by telephone) with the family member that was closest to the patient. Telephone numbers were recorded in the smoking cessation intervention card and also available on the patient’s TB treatment card.

### Definitions and outcome measures

Current smoker was defined as 1) a patient at enrolment who has smoked in the last 3 months, even a puff, and 2) a patient at follow-up visit who has smoked in the last two weeks, even a puff, and has not made any attempt to quit (for at least 24 hours) since the last visit.

Ex-smoker was defined as a patient at enrolment who used to smoke but has not smoked in the last 3 months, not even a puff.

Never smoker was defined as a patient who has never smoked, not even a puff.

Smokefree was defined as zero evidence of smoking: absence of active smoking, the display of “no smoking” signage, and absence of smoking areas/rooms, ashtrays, cigarette butts and the smell of tobacco smoke.

Tobacco-free was defined as zero evidence of smoking and the absence of tobacco advertising, promotion, sponsorship and sales.

Quitter was defined as a smoker at baseline who has not smoked at all, even a puff, in the last 2 weeks at the follow-up visit(s).

Relapsed smoker was defined as a smoker at baseline who has tried to quit during the ABC intervention but has relapsed (has smoked in the last two weeks before the current visit but has made at least one quit attempt lasting at least 24 hours since the last visit).

Lost to follow-up was defined as a patient who did not attend the follow-up visit and whose status was unknown.

Died was defined as a patient who has died of any cause during anti-TB treatment.

## Statistical analysis

Data were analysed using SPSS version 13.0 (Statistical Package for the Social Sciences Inc. Chicago, IL, USA). Differences in frequency distributions were evaluated by the chi square test with p<0.05 as statistically significant. Odds ratio (OR) and 95 % confidence intervals (CI) were estimated using univariable and multivariable binary logistic regression analysis.

### Ethical statement

The Coordinating Committee of Scientific Activity of The Union, the Bogor City Government and City Health Office approved the ABC smoking cessation intervention and its implementation as part of routine TB services. Verbal consent was obtained from each patient and family member that participated in the intervention. Health workers obtained consent from patients to speak with their family members during the follow-up. This study assesses smoking cessation as part of routine services; all information was handled by the health care workers that were providing the routine care for the patients. No individual identifiers were provided to individuals outside the health service.

## Results

### Tobacco-free health care

Of the 80 health care facilities, 52 (65 %) were tobacco-free in March 2011, which increased to 80 (100 %) in December 2012 (Table 1). Smoking was not permitted in any buildings, grounds or carparks. Cigarettes were not sold, and tobacco advertising, promotion and sponsorship were not permitted on the premises.

**Table 1:** Compliance with tobacco-free policy in 80 health facilities, Bogor City March 2011 -December 2012

Indicators	March 2011	October 2011	March 2012	December 2012
	Number (%)	Number (%)	Number (%)	Number (%)
Presence of active smoking	2 (2.5)	1 (1.25)	0 (0)	0 (0)
Presence of smoking area within facility	1 (1.3)	0 (0)	0 (0)	0 (0)
Display of no smoking signage	54 (66.9)	74 (92)	80 (100)	80 (100)
Presence of ashtrays	0 (0)	1 (1.25)	0 (0)	0 (0)
Presence of cigarette butts	22 (27.5)	0 (0)	0 (0)	0 (0)
Presence of any item related to tobacco advertising, promotion or sponsorship	4 (5.0)	0 (0)	2 (2.5)	0 (0)
Presence of cigarette sale at health facility	2 (2.5)	0 (0)	2 (2.5)	0 (0)
Overall compliance to tobacco-free health care policy	52 (65)	73 (91)	78 (98)	80 (100)

### Smoking status and smokefree environments at home at baseline

Of the 750 new smear-positive TB patients registered, 393 (54.4 %) aged 15–34 years, 617 (82.3 %) were male, 582 (77.6 %) were current smokers, 40 (5.3 %) were ex-smokers and 128 (17.1 %) were never smokers. At baseline 139 (18.5 %) had a smokefree home. Males (91.2 %) were significantly more likely to be current smokers as compared to females (14.3 %) (P<0.01); current smokers were less likely to have a smokefree home as compared to ex-smokers and never smokers (p=0.012). Only 12 (1.6 %) patients reported displaying “no smoking” signage at home at baseline (Table 2). Of the 582 current smokers, 199 (34.2 %) usually had their first cigarette within 30 minutes after waking up.

**Table 2:** Characteristics of 750 new sputum smear-positive TB patients, Bogor 2011-2012

Characteristics		Smoking status			
	Total	Current smokers	Ex-smokers	Never smokers	P value
	(N=750)	(N= 582)	(N=40)	(N= 128)	
	n (%)	n (%)	n (%)	n (%)	
Age years^a^					0.053
15-34	393 (54.4)	302 (51.9)	17 (42.5)	74 (57.8)	
35-54	262 (34.9)	214 (36.8)	14 (35.0)	34 (26.6)	
>54	95 (12.7)	66 (11.3)	9 (22.5)	20 (15.6)	
Sex					<0.01
Male	617 (82.3)	563 (96.7)	30 (75.0)	24 (18.8)	
Female	133 (17.7)	19 (3.3)	10 (25.0)	104 (81.2)	
Smokefree home at baseline					0.012
No	611 (81.5)	486 (83.5)	27 (67.5)	98 (76.6)	
Yes	139 (18.5)	96 (16.5)	13 (32.5)	30 (23.4)	
Display of “No smoking” signage at home at baseline					0.172
Signage displayed	12 (1.6)	12 (2.1)	0 (0)	0 (0)	
Signage not displayed	738 (98.4)	570 (97.9)	40 (100)	28 (100)	

^a^median = 33 (minimum 14 and maximum 84), mean 36.62 (SD 14.02)

### ABC in creating smokefree environments at home

**Fig. 2:**
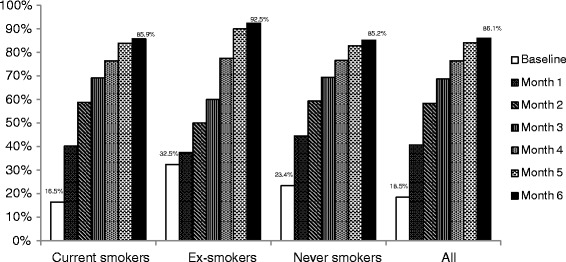
Proportion of the patients with smokefree environments at home at baseline and during monthly follow up

The proportion of current smokers with a smokefree home increased from 16.5 % at baseline to 85.9 % at month six, and that of ex- and never smokers with smokefree homes from 32.5 % to 92.5 % and 23.4 % to 85.2 % respectively (Fig. 2). The proportion of patients displaying”No Smoking” Signage at home increased to 50.8 % at month six from 1.6 % at baseline (Table 3).

**Table 3:** Display of “No smoking” signage at home among 750 TB patients, at baseline and at six months

Smoking status	Total	Display No smoking signage at home at baseline and after ABC	
	n	At baseline	At end of six months
		n (%)	n (%)
Current smokers	582	12 (2.1)	276 (47.4)
Ex-smokers	40	0 (0)	20 (50.0)
Never smokers	128	0 (0)	85 (66.4)
All	750	12 (1.6)	381(50.8)

### ABC for smoking cessation

Table 4 presents the outcomes of the ABC smoking cessation intervention. The point prevalence of the quit rate was 41.1 % at month 1. It increased substantially to 66.8 % at month six. Twenty-one self-reported quitters were identified as relapsed smokers after verification with a family member at month six. They were re-classified as relapsed smokers throughout the analysis.

**Table 4:** Point prevalence outcomes of ABC intervention among 582 current smokers with TB at baseline

Outcome	Outcomes based on health staff asking patients about smoking status at monthly visits and on TB registers					
	Month 1	Month 2	Month 3	Month 4	Month 5	Month 6
	N (%)	N (%)	N (%)	N (%)	N (%)	N (%)
Quitter	239 (41.1)	329 (56.5)	366 (62.9)	388 (66.7)	406 (69.8)	389 (66.8)
Relapsed smoker	37 (6.4)	56 (9.6)	74 (12.7)	70 (12.0)	54 (9.3)	62 (10.7)
Died	2 (0.3)	5 (0.9)	7 (1.2)	11 (1.9)	15 (2.6)	15 (2.6)
Lost to follow up	17 (2.9)	37 (6.4)	39 (6.7)	51 (8.8)	56 (9.6)	71 (12.2)
Current smoker	287 (49.3)	155 (26.6)	96 (16.5)	62 (10.7)	51 (8.8)	45 (7.7)
Total	582 (100)	582 (100)	582 (100)	582 (100)	582 (100)	582 (100)

Of the 389 quitters at month six, 82.3 % had observed three months of continuous abstinence, 11.8 % two months of continuous abstinence and 5.9 % two weeks of continuous abstinence.

In multivariable logistic regression analysis (Table 5) three predictors remained independently associated with quitting: a time from waking to first cigarette of >30 minutes, having a smokefree home and the display of “no smoking” signage at home at month six.

**Table 5:** Multiple logistic regression analysis of factors associated with quitters

Characteristics	Smoking status at month 6 after ABC intervention			
	Patients (N= 582)	Quitters n (%)	Odds ratio (OR) 95 % Confidence interval (CI)	Adjusted OR 95 % CI
Age, years				
15-34 (reference)	302	204 (67.5)	1.00	1.00
35-54	214	143 (66.8)	1.19 (0.68-2.07)	1.07 (0.54-2.10)
>54	66	42 (63.6)	1.15 (0.64-2.04)	0.98 (0.49-1.97)
Sex				
Female (reference)	19	12 (63.2)	1.00	1.00
Male	563	377 (67.0)	1.18 (0.46-3.05)	1.48 (0.48-4.57)
Time from waking to first cigarette				
≤30 minutes (reference)	199	96 (48.2)	1.00	1.00
>30 minutes	383	293 (76.5)	3.49 (2.43-5.03)	3.14 (2.05-4.81)
Smokefree environments at home after ABC intervention				
No (reference)	82	20 (24.4)	1.00	1.00
Yes	500	369 (73.8)	8.73 (5.08-15.01)	3.23 (1.79-5.82)
Display of “no smoking” signage at home after ABC intervention				
No (reference)	306	141 (46.1)	1.00	1.00
Yes	276	248 (89.9)	10.37 (6.60-16.27)	7.75 (4.78-12.57)

## Discussion

The ABC smoking cessation intervention was effective for: i) creating 100 % tobacco-free health services, ii) promoting quitting smoking (66.8 %) and iii) establishing smokefree environments at home (86.1 %). The high quit rates in our study are probably due to several factors, such as: i) the enabling environments in a health system that offers 100 % tobacco-free health services; ii) routine screening for smoking and smokefree homes; iii) brief advice to quit smoking and to create smokefree homes; iv) cessation support with additional information on the danger of tobacco smoking and secondhand smoke, and v) regular reminders and encouragement of family members to quit smoking. A similar intervention piloted in Bangladesh and India also reported high quit rates of tobacco use (Bangladesh 82 % and India 67.3 %). These studies identified brief advice through regular DOTS services as an effective intervention to promote quitting tobacco use [11, 12]. Guidelines for implementation of article 14 of the WHO Framework Convention on Tobacco Control recommend brief advice to stop using tobacco, usually taking only a few minutes, given to all tobacco users, usually during the course of a routine consultation or interaction [13].

Smoke-free environments encourage people to quit smoking and reduce the likelihood of exposure to secondhand smoke. Our study demonstrates that a smokefree environment at home is strongly associated with quitters, indicating that banning smoking inside the home might be a highly motivating act that convinces smokers to quit smoking and quitters to remain quitters. No smoking signage was used to conveying the message to family members and visitors to the home that smoking is prohibited. Therefore, our study suggests that smoking cessation counselling together with providing tobacco-free environments both at the health care facility and at home may produce a large public health impact.

Published evidence suggests that exposure to secondhand smoke increases the risk of developing a range of illnesses including lung cancer, heart disease, stroke, respiratory illness, sudden infant death syndrome (SIDS), ear infection, and severe asthma in children, pneumonia, and low birth weight [14]. We found 76.6 % (98/128) of the never smokers with TB were exposed to secondhand smoke at home at baseline, indicating that scaling up the creation of smokefree environments at home may contribute to TB control because exposure to respirable pollutants due to the combustion of tobacco increases the risk of both TB infection and TB disease [15].

Most research identified that tobacco smoking increases the risk of developing TB, delay in TB diagnosis, worse treatment outcomes and relapse [5, 16]. Smokers who quit reduce both their risk of becoming infected with TB and their risk of dying from it. One study showed TB mortality dropping by almost two-thirds for quitters, compared to those who continued to smoke, and the risk for quitters almost returned to the level of those who had never smoked [17].Thus, prompt identification of smoking behaviour and smoking cessation are critical for improving TB treatment and reducing transmission of TB.

Our study has several strengths. Smokefree environments in all public places and work places have been established in Bogor city since 2010. The involvement of local health staff right from the beginning of the intervention has helped to sustain the smoking cessation care and tobacco-free environments in primary health services. All health centres were located in the community, so it was straightforward for health staff to conduct random inspections in patients’ homes and to have informal interactions with patients to monitor progress. The study does, however, have some possible limitations. The outcomes of the intervention were largely evaluated on the basis of self-reporting. There might be a bias in the responses of the patients, in that they might have said what they thought the health worker would want to hear, thus overestimating the effect of the intervention. However, we have validated the status of the patients’ smoking and of their smokefree home with a family member at month six. Issues of time constraints and high workload were raised at the initial training by the health staff before the intervention; however, health care workers reported neither high workloads nor time constraints during the review meeting at month two, five and six.

## Conclusions

Our study has demonstrated the effectiveness of the ‘ABC’ smoking cessation intervention, which was delivered in just 5 to 10 minutes by health staff as a part of existing DOTS services. The intervention resulted in high quit rates and higher awareness of the adverse health effects of secondhand smoke, which led patients to make smoke-free homes and health providers to make tobacco-free health care. The study also proved that ABC is feasible in DOTS services and primary health care in Indonesia. We believe that ABC can and should be generalised to other services and levels of care in Indonesia. It can be integrated into general primary health care services to enhance the impact of public health programmes, through implementing effective measures of tobacco control such as smoke-free environments, banning tobacco advertising, promotion and sponsorship and offering help to quit tobacco use.

### What this paper adds to

Ingredients to produce high quit rates are:

- Creating smokefree environments in health services
- **Offering A**sk, **B**rief advice with minimal **C**essation support by health workers at each visit
- Engaging family members to support patients to quit smoking
- Providing additional information on the dangers of smoking and secondhand smoke
- Encouraging patients and family members to create smoke-free environments at home
- Providing No smoking signage to display at home
- Monitoring the status of the intervention through face-to-face interaction with patients and family members

## References

[1] Maurya V, Vijayan K, Shah A (2002). Smoking and tuberculosis: an association overlooked. Int J Tuberc Lung Dis.

[2] Gajalakshmi V, Peto R, Kanaka TS, Jha P (2003). Smoking and mortality from tuberculosis and other diseases in India: retrospective study of 43000 adults male deaths and 35000 controls. Lancet.

[3] Sun Ha J, Jonathan EG, Jaeseong J, II Su P, Heechoul O, Jonathan MS (2009). Smoking and risk of tuberculosis incidence, mortality and recurrence in South Korean Men and Women. Am J Epidemiol.

[4] World Health Organization: Tuberculosis and Tobacco. World Health Organization, Geneva. http://www.who.int/tobacco/publications/health_effects/factsheet_tub_tob.pdf?ua=1. Accessed 31 December 2014.

[5] World Health Organization (2007). A WHO/The Union Monograph on TB and Tobacco Control: joining efforts to control two related global epidemics.

[6] Bissell K, Fraser T, Bam TS (2011). World No Tobacco Day: from an international treaty to country level action. Int J Tuberc Lung Dis.

[7] Bissell K, Fraser T, Chiang C-Y, Enarson DA: Smoking cessation and smokefree environments for tuberculosis patients. Paris, France: International Union Against Tuberculosis and Lung Disease, 2010. http://www.theunion.org/what-we-do/publications/technical/smoking-cessation-and-smokefree-environments-for-tuberculosis-patients. Accessed 2 January 2015.

[8] World Health Organization: Global Tuberculosis Report 2013. WHO, Geneva. www.who.int/iris/bitstream/10665/91355/1/9789241564656_eng.pdf. Accessed 5 December 2014.

[9] World Health Organization Regional Office for South East Asia: Global Adult Tobacco Survey: Indonesia Report 2011. New Delhi, India: WHO, 2012. http://www.who.int/tobacco/surveillance/survey/gats/indonesia_report.pdf. Accessed 5 December 2014.

[10] United Nations, Department of Economic and Social Welfare: World Population Prospects: The 2012 Revision http://worldpopulationreview.com/countries/indonesia-population/major-cities-in-indonesia/. Accessed 10 December 2014.

[11] Siddiquea BN, Islam MA, Bam TS, Satyanarayana S, Enarson DA, Reid AJ (2013). High quit rate among smokers with tuberculosis in a modified smoking cessation programme in Dhaka, Bangladesh. PHA.

[12] Kaur J, Sachdeva KS, Modi B, Jain DC, Chauhan LS, Dave P (2013). Promoting tobacco cessation by integrating ‘brief advice’ in tuberculosis control programme. WHO South-East Asia J Public Health.

[13] World Health Organization: Guidelines for Implementation of article 14 of the WHO Framework Convention on Tobacco Control. Geneva: WHO 2010. http://www.who.int/fctc/guidelines/adopted/article_14/en/. Accessed 10 December 2014.

[14] U.S. Department of Health and Human Services. The Health Consequences of Involuntary Exposure to Tobacco Smoke: A Report of the Surgeon General. Atlanta, Georgia: U.S. Department of Health and Human Services, Centers for Disease Control and Prevention, Coordinating Center for Health Promotion, National Center for Chronic Disease Prevention and Health Promotion, Office on Smoking and Health. 2006.

[15] Lin HH, Ezzati M, Murray M (2007). Tobacco Smoke, Indoor Air Pollution and Tuberculosis: A Systematic Review and Meta-Analysis. PLoS Med.

[16] Bam TS, Enarson DA, Hinderaker SG, Bam DS: Longer delay in accessing treatment among current smokers with new sputum smear-positive tuberculosis in Nepal. Int J Tuberc Lung Dis 2012;16(6):822–7. http://www.ncbi.nlm.nih.gov/pubmed/22507563. Accessed 3 November 2014. 10.5588/ijtld.11.0678 22507563

[17] Wen C-P (2010). The reduction of tuberculosis risks by smoking cessation. BMC Infect Dis.

